# A Design-assisted Spectrofluorometric Method Utilizing a One-pot Fluorescent Probe for the Quantitation of some Calcium Channel Blockers

**DOI:** 10.1007/s10895-022-03089-9

**Published:** 2022-12-08

**Authors:** Aya Roshdy, Randa Abdel Salam, Ghada Hadad, Fathallah Belal, Heba Elmansi

**Affiliations:** 1 Department of Pharmaceutical Chemistry, Faculty of Pharmacy, Horus University-Egypt, New Damietta, Egypt; 2 Department of Pharmaceutical Chemistry, Faculty of Pharmacy, Suez Canal University, Ismailia, Egypt; 3 Department of Pharmaceutical Analytical Chemistry, Faculty of Pharmacy, Mansoura University, Mansoura, 35516 Egypt

**Keywords:** Quantum Dots, Calcium Channel Blockers, Inner Filter Effect, Experimental Design

## Abstract

Based on their reaction with highly fluorescent carbon quantum dots (CQDts), a precise and reliable spectrofluorometric approach was developed for the determination of three calcium channel blockers. The studied drugs are: lercanidipine, nimodipine and nifedipine. (CQDts) were produced using a one-step hydrothermal method with ascorbic acid as the carbon source. The produced CQDts were capped by alcohol to create yellow emitters displaying a high fluorescence emission at 524 nm when excited at 325 nm. The fluorescence intensity of CQDts was noticeably quenched by each of the three calcium channel blockers. The relation between their concentrations and fluorescence quenching is linear over the concentration range of 0.5–20 µg/mL for each of the three drugs. A full factorial design was used to optimize the effect of variable factors. Therefore, under optimum experimental design conditions, the detection limits for lercanidipine, nimodipine, and nifedipine were 0.11 ± 1.09, 0.10 ± 0.25 and 0.12 ± 0.71 µg/mL, respectively. The LOQ was 0.33, 0.30, and 0.37 µg/mL respectively. The quenching of fluorescent CQDts occurred through the inner filter effect (IFE) for nimodipine, while it was mixed with dynamic quenching for lercanidipine and nifedipine. The proposed method was effectively used to determine the cited drugs in their pharmaceutical products and had an acceptable level of precision. The selectivity of the CQDts system towards the studied drugs was examined indicating no interference from interfering species.

**Supplementary Information:**

The online version contains supplementary material available at 10.1007/s10895-022-03089-9.

## Introduction

Hypertension is a leading risk factor for the development of cardiovascular diseases and is increasing in prevalence. Despite improvements in knowledge and care, hypertension continues to be the leading cause of morbidity and mortality, accounting for the greatest number of combined years of life lost and years lived with disability [1].

A class of drugs called calcium channel blockers (CCBs) is used to treat hypertension. They function by inhibiting a portion of the calcium that reaches the arteries and heart [2]. This work deals with three commonly described (CCBs) including lercanidipine, nimodipine, and nifedipine.

**Fig. 1:**
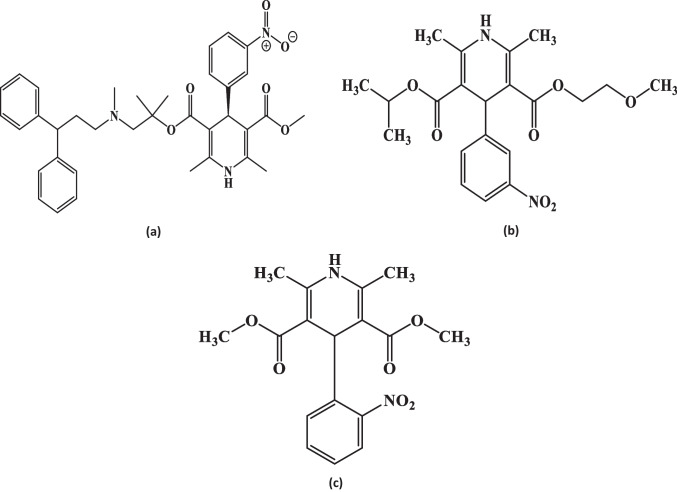
Structural formulae of Lercanidipine, Nimodipine and Nifedipine. **a** Lercanidipine **b** Nimodipine **c** Nifedipine

Lercanidipine (Fig. 1a), is 2-[(3,3-diphenylpropyl) methylamine]-1,1- dimethylethylmethyl1,4-dihydro-2,6-dimethyl-4-(3- nitrophenyl)-3,5pyridinedicarboxylic ester [3].

Different analytical techniques, such as spectrophotometry [4–7] and chromatography [8–11] have been used for their determination.

Nimodipine (Fig. 1b), chemically is 2-Methoxyethyl 1-methyl ethyl (4RS)-2,6-dimethyl-4- (3-nitrophenyl)-,4-dihydropyridine-3,5-dicarboxylate [12]. Numerous analytical techniques, such as spectrophotometry [13–16], spectrofluorimetric [17], and chromatographic [18–21] were reported for its determination.

Nifedipine (Fig. 1c), chemically is dimethyl 2,6-dimethyl-4-(2-nitrophenyl)-1 4- dihydropyridine-3,5-dicarboxylate [12]. Various analytical techniques have been reported for the determination of nifedipine including spectrophotometry [22–25], spectrofluorimetric [26, 27], and chromatography [28–30].

The proposed method has the benefit of being more sensitive than the reported spectrofluorimetric methods as the linearity range was 0.5–20 µg/mL for the studied drugs. In addition, it was used with minimum amounts of organic solvents compared to HPLC methods.

Recently, carbon Quantum dots (CQDts) have been distinguished as the top choice among a variety of fluorescent materials because of their interesting characteristics, which include exceptional biocompatibility, potent fluorescence capabilities, and simple preparation [31].

carbon Quantum dots (CQDts) are nanoparticles with size-dependent optical and electrical characteristics that have been presented for a variety of applications due to their high extinction coefficients and cost–benefit analysis. CQDts have rapidly found use in bioimaging [32] photovoltaic technology [33] and nanomedicine [34]. CQDts have been already investigated for the spectroscopic determination of several pharmaceuticals including amoxicillin, vitamin B12 histidine, and tetracycline [35].

Experimental design is a logical process of planning experiments with enough statistical power, sample size, and data type to get the maximum amount of information from chemical data and effectively handle the limitations and objectives set by the planned research. These multivariate statistical methods have benefits, including a lower number of experiments required, and consequently lower reagent consumption, less laboratory work, and the ability to develop mathematical models that allow evaluation of the importance and statistical significance of the communication effects between factors [36].

The current work focuses on the optimization, development, and validation of a reliable spectrofluorimetric approach for the analysis of three (CCBs) in their dosage forms using experimental design.

## Experimental

### Apparatus

- Cary Eclipse fluorescence spectrophotometer equipped with Xenon flash lamp from Agilent Technologies was used at 800 Volt. The excitation and emission wavelengths were 325/528 nm. A smoothing factor of 20 was used.
- Shimadzu UV- Visible 1601 recording Spectrophotometer (P/N 206–67,001). Recording range: 0–1.0.
- Minitab^®^ Statistical Software was used to perform the factorial design and statistical analysis (release 16 for windows, state college, Pennsylvania).
- A Consort NV P-901 pH –Meter (Belgium) was used for pH measurements
- Fourier transform infrared (FT-IR) spectroscopy spectra were conducted on an IS10 Nicolet spectrophotometer (USA).
- Transmission electron microscopy (TEM) images of nanoparticles were acquired on a JSM-2100 transmission electron microscope (JEOL, Japan) operating at 200 kV. All optical measurements were performed at room temperature.

### Materials and Reagents

- Ascorbic acid and Ethylene glycol, sodium acetate, and acetic acid were purchased from El-Nasr Pharmaceutical Chemicals Company (ADWIC), Egypt, and were used without further purification.
- Lercanidipine (99.7% purity) was kindly supplied by Multi-Care Company for Pharmaceuticals, Cairo, Egypt.
- Nimodipine and Nifedipine (99.9% purity) were obtained from National Organization for Drug Control and Research, (NODCAR), Giza, Egypt.
- Different dosage forms were obtained from commercial pharmacies in the local market, including:
- Caredipine^®^ tablets labeled to contain 10 mg Lercanidipine Hydrochloride, a product of Multi Care Company for Pharmaceuticals, Cairo, Egypt. Batch number 112836.
- Nimodipine^®^ tablets labeled to contain 30 mg Nimodipine Dihydropyridine, a product of Pharco pharmaceuticals, Alexandria, Egypt. Batch number 10210.
- Epilat Retard^®^ tablets labeled to contain 20 mg Nifedipine, a product of Egypt International Pharmaceutical Industries CO Company, 10th Ramadan City, Egypt. Batch number 2100993.

### Standard Solutions

A stock solution from each drug was prepared separately in a 100 mL volumetric flask by carefully weighing 0.010 g of each drug then dissolving and completing to the mark with methanol to reach a concentration of 100.0 μg/mL. The stock solution of the studied drugs was freshly prepared and kept away from the light.

### Preparation of buffer

Acetate buffer solution 0.2 M (pH ranges from 4 to 6), was prepared by mixing suitable volumes of 0.2 M sodium acetate and 0.2 M acetic acid and adjusting the pH using the pH meter.

### CQDts Synthesis

They were synthesized using the hydrothermal one-pot method [31], which involved dissolving ascorbic acid (0.8 g) in a solution of water and ethylene glycol (20 mL) (volume ratio 1:1). After vigorous stirring to create a homogeneous solution, the liquid was heated to 120° C in 6 h. The dark yellow color arises from the colorless solution. The solution produced was cooled to room temperature and centrifuged at 12,000 rpm for 10 min. Dual emitting CQDts were produced and kept at 4 °C until used.

### General Procedure

#### Construction of the Calibration Curves

A 1:1 dilution of the dual emitting CQDts in water was followed by the addition of 0.5 mL of the diluted CQDts to a 10 mL volumetric flask. In a separate set of 10 mL volumetric flasks, different known aliquots of the working standard solution of lercanidipine, nimodipine, and nifedipine (0.5–20.0 µg/mL) were added followed by 2 mL of acetate buffer of pH 6. Finally, distilled water was added to complete the volume to the mark, thoroughly mixed, and left to stand for 15 min. Yellow emitters' fluorescence was observed at a wavelength of 528 nm upon excitation at 325 nm. Similarly, a blank experiment was performed. The calibration graph for each of the studied drugs was created by plotting the quenching value (ΔF) against the drug concentrations. The regression equations were then derived.

#### Determination of the studied drugs in tablets

Ten tablets of each of Caredipine^®^, Nimodipine^®^, and Epilat Retard^®^ tablets were weighed, ground, and finely powdered. After blending a weight of the fine powder equal to 10.0 mg was transferred into a 100 ml volumetric flask with about 50 mL of methanol, and sonicated for 20 min before being completed with 100.0 mL of methanol. The solution required sonication again for 20.0 min. Filter: the first portion of the filtrate was rejected as the solution was filtered. By serially diluting the filtrate, varied concentrations covering each drug’s concentration range were analyzed. The procedure described under "Construction of the Calibration Curves" was then applied and the nominal content of each drug in its tablets was calculated using the corresponding regression equation.

## Results and Discussion

Carbon-based nanomaterials have received a lot of interest in recent years. Carbon quantum dots (CQDts), novel zero-dimensional nanomaterials based on carbon, are well-known for their small size and comparatively strong fluorescence characteristics. CQDts not only inherit the excellent optical properties of traditional semiconductor quantum dots but also overcome their shortcomings in terms of cytotoxicity, environmental danger, and biohazard by having superior water solubility, chemical stability, and photobleaching resistance. It has caused tremendous anxiety among researchers in several fields, including biology, chemical sensing, nanomedicine, and photo electrocatalysis [37].

CQDts were created from ascorbic acid as a carbon source. It was dissolved in water and subjected to hydrothermal carbonization. Alcohol (polyethylene glycol) added in excess formed the ligand and capped the CQDts, producing yellow emitters. The ligand groups on the surface of the CQDts were visible in the FT-IR spectrum (Fig. S1). The peak at 3383 cm-1 denotes the presence of the O–H group, while the peaks at 2943 and 1655 cm^−1^ denote the stretching vibrations of the C-H and C = O, respectively, and the peak at 1086 cm^−1^ denotes the symmetric and asymmetric vibration of the C–O–C [38]. Because of the hydroxyl and carbonyl groups, CQDts have strong water solubility.

**Fig. 2:**
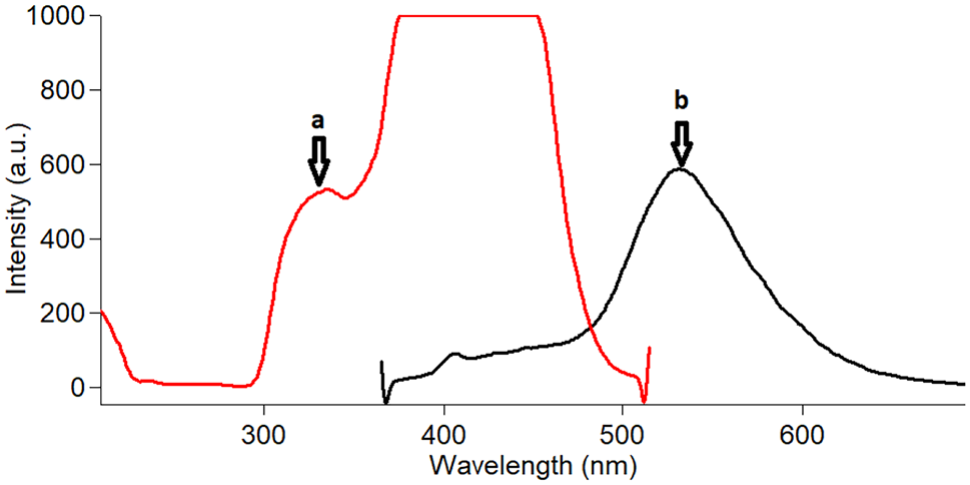
Excitation and emission spectra of CQDs where a: for excitation at 325 and b: for emission at 528

The aqueous excitation-emission spectra are presented in Fig. 2 at wavelengths of 325 nm and 524 nm, respectively. The average diameter of the spherical CQDts shape in the transmission electron microscopy (TEM) image was 10 nm (Fig. S2).

### Optimization of Experimental Factors

To examine all potential combinations of the variables and their influence on various responses, the procedure optimization was conducted using a two-level full factorial design. The factorial design has distinct advantages over the conventional optimization method, including the use of fewer experiments, shorter operation times, and the ability to generate data that can be statistically analyzed to reveal important details about the interactions between experimental parameters [39]. A total of 18 experiments were conducted to determine the effects of time, buffer volume, buffer pH, and reagent volume on the ΔF for the three drugs. The factors and ranges selected for consideration were based on previous univariate studies which show the best quenching when the range of volume of the reagent was 0.2 to 0.5 mL (A), pH of the buffer was 4 to 6 (B) and the volume of buffer was 2 to 6 mL (C). After the addition of the reagent solution to the drugs, different time intervals were studied from zero to 15 min (D).

Half-normal plots and Pareto charts generated by the factorial design are shown in Fig. S3 demonstrating that the volume of the reagent (A) had a significant effect on the ΔF of each of the three drugs. Additionally, the volume of the reagent with the buffer's pH (AB) had a significant effect on the ΔF of only nimodipine and nifedipine, whereas the volume of the buffer (C) had a significant effect on the ΔF of lercanidipine alone.

To investigate these independent factors and their interactions that affect the responses, an approximated Fisher Statistical Test for Variance Analysis (ANOVA) model [40] was employed to analyze the responses and used to determine the significance of the independent factors.

The equation for the four-factor experimental design could be presented as follows:

$$\upgamma = {\upbeta }_{0}+{\upbeta }_{1}\mathrm{A}+{\upbeta }_{2}\mathrm{B}+{\upbeta }_{3}\mathrm{C}+{\upbeta }_{4}\mathrm{D}+{\upbeta }_{2}\mathrm{AB}+{\upbeta }_{2}\mathrm{AC}+{\upbeta }_{2}\mathrm{BC}+{\upbeta }_{2}\mathrm{BD}+{\upbeta }_{2}{\mathrm{A}}^{2}+{\upbeta }_{2}{\mathrm{B}}^{2}+{\upbeta }_{2}{\mathrm{C}}^{2}+{\upbeta }_{2}{\mathrm{D}}^{2}$$

where:

γ: stands for a response
β: stands for regression coefficients, and
A, B, C, and D: stand for quantum dot reagent, buffer pH, the volume of buffer, and time of incubation, respectively.

The interaction between different factors was confirmed by the interaction and main effect plots (Fig. S4) which showed that there are interactions between the factors affecting the method and these can’t be shown using univariate optimization.

**Fig. 3:**
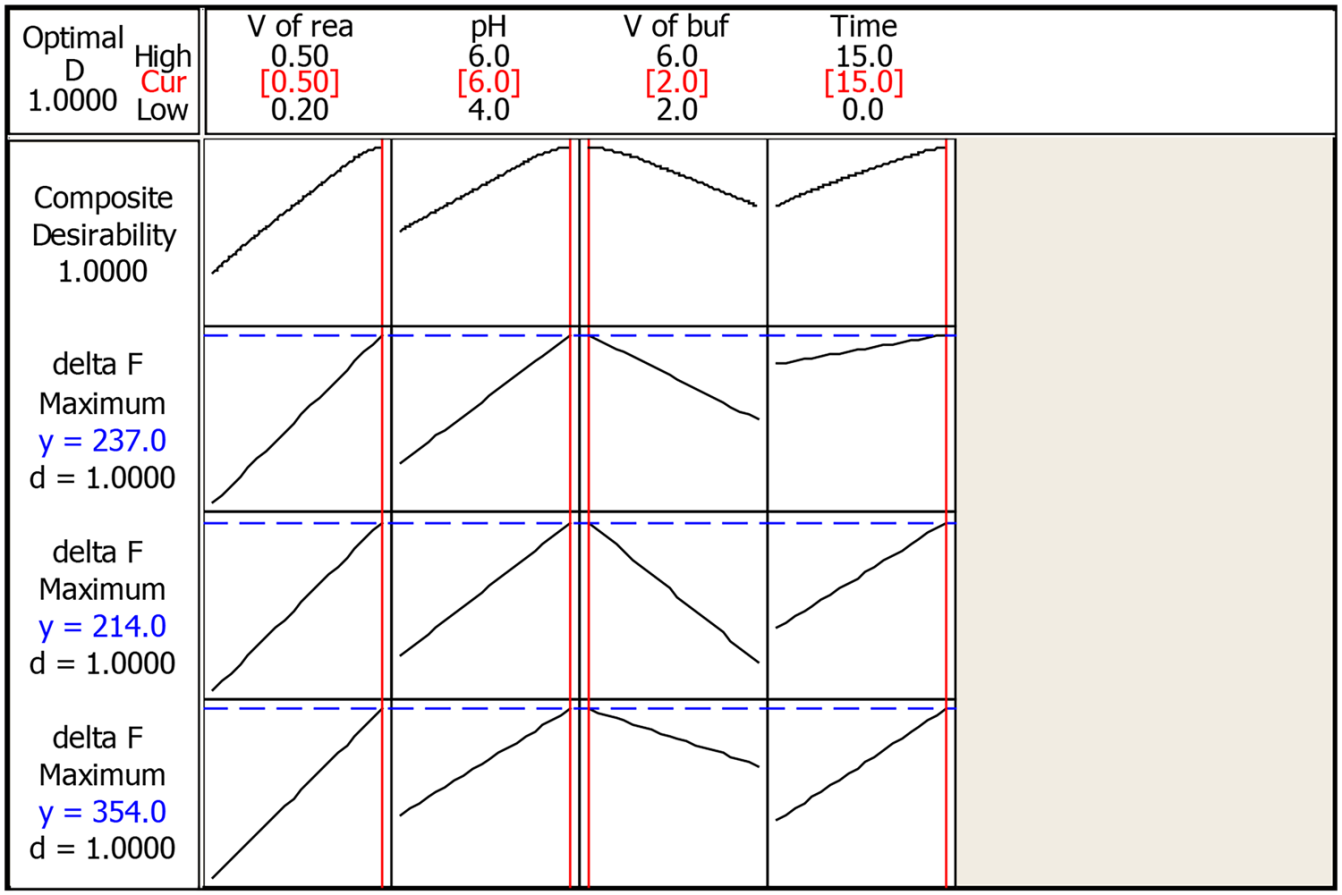
2^3^ FFD optimization plot

The composite desirability of response is determined by the Minitab response optimizer. The value of (D), which ranges from zero to one, indicates if the responses fall within acceptable limits. One implies that the condition attained is ideal, hence its value should be one or near to one. Zero is unacceptable since it shows that many of the responses are outside of their acceptable ranges. It provided the response optimizer (Fig. 3) which shows that the optimum conditions to achieve the highest ΔF when the volume of reagent was 0.5, pH was 6, the volume of buffer was 2 mL and the incubation time was 15 min.

### Comparison with Previous Methods

The proposed method is the first spectrofluorimetric method for the determination of lercanidipine. Furthermore, the reported spectrofluorimetric methods for the determination of nimodipine (17) and nifedipine (26–27) are time-consuming and need prior chemical derivatization, while the proposed study is considered green with a minimal amount of organic solvents, simple, direct and more sensitive with wider ranges for both drugs.

The concept of measurements is based on the quenching of QDs that are simply synthesized using ascorbic acid and alcohol with water to generate high fluorescent carbon QDs.

Additionally, the optimization was performed using a full factorial design which enables the analysis of the data and reduces the time required for the experiment.

### Analytical Performance of the Proposed Method

The suggested technique was approved according to ICH Q2 recommendations [41]. For each of the three drugs, the fluorescence intensity was quenched linearly in the range of 0.5–20.0 µg/mL. Linear regression analysis of the data is summarized in Table 1, and represented by the following equations:

**Table 1:** Performance data of the studied drugs by the proposed methods

**Parameter**	**Lercanidipine**	**Nimodipine**	**Nifedipine**
**concentration range(µg/ mL)**	0.5–20.0	0.5–20.0	0.5–20.0
**-LOD (µg/mL)**	0.11	0.10	0.12
**-LOQ (µg/ mL)**	0.33	0.30	0.37
**-Correlation coefficient (r)**	0.9999	0.9999	0.9999
**-Slope**	23.38	18.58	21.14
**-Intercept**	38.14	24.22	117.73
**-Sy/x**	1.1097	0.8131	1.215
**-Sa**	0.7718	0.541	0.845
**-Sb**	0.0675	0.0462	0.074
**-% Error**	0.445	0.100	0.29
**-%RSD**	1.090	0.246	0.712
**-No.of Experiments**	6	6	6
**-Mean found (%) ± SD**	100.04 ± 1.09	99.95 ± 0.25	99.93 ± 0.71

N.B

-S_y/x_ = standard deviation of the residuals

-S_a_ = standard deviation of the intercept of the regression line

-S_b_ = standard deviation of the slope of the regression line

-% Error = RSD% / √ n

$$\begin{array}{ccc}\Delta \mathrm{F }= 38.41+23.38\mathrm{C}& (\mathrm{r}=0.9999)& \mathrm{for lercanidipine}\\ \Delta \mathrm{F }= 24.22+18.58\mathrm{C}& (\mathrm{r}=0.9999)& \mathrm{for nimodipine}\\ \Delta \mathrm{F }= 117.37+21.14\mathrm{C}& (\mathrm{r}=0.9999)& \mathrm{for nifedipine}\end{array}$$

where ∆F is the quenching in the fluorescence intensity, ∆F = (the native fluorescence of quantum dot solution (Fº)—fluorescence of the reaction product (F)), C is the concentration of the drug (μg/mL) and r is the correlation coefficient.

The limits of quantitation (LOQ) were calculated following ICH Q2 Recommendations [41[ and were found to be 0.33 for lercanidipine, 0.30 for nimodipine, and 0.37 μg/mL for nifedipine. LOQ was calculated from the following equation [41]:

$$\mathrm{LOQ }= 10 {\mathrm{S}}_{\mathrm{a}} /\mathrm{ slope}$$

The limits of detection (LOD) were calculated accordingly and were found to be 0.11 ± 1.09 for lercanidipine, 0.10 ± 0.25 for nimodipine and 0.12 ± 0.71 μg/mL for nifedipine. LOD was calculated from the following equation [41].

$$\mathrm{LOD }= 3.3 {\mathrm{S}}_{\mathrm{a}} /\mathrm{ slope}$$

The proposed methods were tested for linearity, specificity, accuracy, and precision (Table 1).

For examining precision, three concentrations of each drug (5.0,10.0,15.0 µg/mL) in its pure form on three separate occasions were tested, and the results are displayed in (Table 2). The drugs were repeatedly studied in pure form using different concentrations summarized in (Table 2) over a period of three days to achieve intermediate precision (also known as ruggedness). Moreover, the consistency of the difference in fluorescence intensity (ΔF) with the slight changes in the experimental parameters proved the method^’^s robustness. These parameters included pH 6 ± 0.2, the volume of buffer 2 ± 0.2, and the volume of the quantum dot reagent 0.5 ± 0.02. These minimal changes that may take place during the experimental operation don’t greatly affect the decrease in the fluorescence intensity (∆F).

**Table 2:** Precision data of the proposed methods for determination of the studied drugs in pure form

**Drug**	**Conc. (μg/mL)**	**Intra-day**			**Inter-day**		
		**Mean ± S. D**	**%RSD**	**% error**	**Mean ± S. D**	**%RSD**	**% error**
**Lercanidipine**	5	99.17 ± 0.99	1.00	0.58	99.35 ± 1.03	1.04	0.60
	10	99.95 ± 0.42	0.42	0.24	99.59 ± 0.91	0.92	0.24
	15	99.97 ± 0.60	0.60	0.34	99.78 ± 0.73	0.73	0.42
**Nimodipine**	5	99.67 ± 0.54	0.54	0.31	99.81 ± 0.37	0.37	0.21
	10	99.98 ± 0.59	0.59	0.34	99.65 ± 1.13	1.14	0.66
	15	99.55 ± 0.94	0.95	0.55	99.75 ± 0.66	0.66	0.38
**Nifedipine**	5	99.68 ± 0.61	0.61	0.78	99.4 ± 0.8	0.83	0.44
	10	100.4 ± 0.9	0.92	0.53	99.8 ± 0.5	0.51	0.29
	15	99.6 ± 0.6	0.74	0.43	100.25 ± 0.35	0.36	0.21

The proposed and comparison methods for each of the three drugs did not show significant differences came to accuracy and precision, according to statistical analysis of the results using the Student's t-test and variance ratio F-test [42].

The comparison techniques used spectrophotometric measurements of the three drugs' absorbances in their solutions at wavelengths of 332 nm, 238.5 nm, and 387 nm for lercanidipine, nimodipine, and nifedipine, respectively [5, 13, 25].

The proposed methods are simple, fast, sensitive, and have a wider range than the comparison method.

## Pharmaceutical Applications

The proposed method was effectively utilized in the determination of each of the three drugs in their dosage forms Caredipine^®^, Nimodipine^®^, and Epilat Retard^®^ tablets.

By observing any interference caused by common tablet excipients such as talc, lactose, starch, avisil, gelatin, and magnesium stearate, the method's specificity was examined. The acceptable % recoveries of the examined concentrations showed that these excipients didn't interfere with the suggested approach. The results of the analysis of tablets and in pure form using the suggested method are shown in (Table 3). Comparison with the spectrophotometric approach previously reported, shows good accuracy and precision.

**Table 3:** Application of the proposed method and reference method for determination of the studied drugs in commercial tablets

**Compound**	**Proposed Method**			**Comparison methods **[5, 13, 25]
	**Amount taken (µg/mL)**	**Amount found (µg/mL)**	**% Found**	**% Found**
**Caredipine**	5	4.920	98.40	100.92
	10	9.898	98.98	99.09
	15	14.932	99.55	100.31
**Mean**	98.98			100.11
** ± S.D**	0.575			0.93
**t-test**	1.81(2.78)			
**F-test**	2.62(19.00)			
**Nimodipine**	5	4.991	99.83	100.79
	10	9.857	98.57	99.28
	15	15.054	100.36	100.29
**Mean**	99.59			100.12
** ± S.D**	0.919			0.77
**t-test**	0. 83(2.78)			
**F-test**	1.42(19.00)			
**Epilat retard**	5	5.0100	100.21	100.47
	10	9.9240	99.24	98.89
	15	15.0400	100.27	99.93
**Mean**	99.91			99.76
** ± S.D**	0.578			0.80
**t-test**	0.27(2.78)			
**F-test**	1.92(19.00)			
**Caredipine**	5	4.920	98.40	100.92
	10	9.898	98.98	99.09
	15	14.932	99.55	100.31
**Mean**	98.98			100.11
** ± S.D**	0.575			0.93
**t-test**	1.81(2.78)			
**F-test**	2.62(19.00)			
**Nimodipine**	5	4.991	99.83	100.79
	10	9.857	98.57	99.28
	15	15.054	100.36	100.29
**Mean**	99.59			100.12
** ± S.D**	0.919			0.77
**t-test**	0. 83(2.78)			
**F-test**	1.42(19.00)			
**Epilat retard**	5	5.010	100.21	100.47
	10	9.924	99.24	98.89
	15	15.040	100.27	99.93
**Mean**	99.91			99.76
** ± S.D**	0.578			0.80
**t-test**	0.27(2.78)			
**F-test**	1.92(19.00)			

Values between parentheses are the tabulated t and F values respectively, at p = 0.05. [42]

### Study of Interference

The possible interference from other species that might be present was examined to assess the analytical method's selectivity. Taking into account that the target samples to be analyzed are CCBs, the main compounds that may interfere in the determination are other antihypertensive drugs such as atenolol [6], captopril, atorvastatin, valsartan, clopidogrel, metformin, albendazole, rupatadine, miconazole, gabapentin, and ezetimibe.

Other interfering agents were introduced to examine the selectivity of these agents including ascorbic acid, uric acid, magnesium sulfate, and copper sulfate.

If the signal variation was less than 5%, we assumed that no interference had occurred. In general, the tolerance to the presence of foreign species is much higher than the concentration at which these compounds are often present with the analytes in pharmaceuticals (Table 4). Satisfactory tolerances were obtained due to the selectivity of the CQDts system towards the studied drugs, observing no interaction with other antihypertensive drugs and interfering agents.

**Table 4:** Study of interference of excipients

**Interfering substance**	Tolerance limit *
**Atenolol**	11.50
**Captopril**	20.00
**Atorvastatin**	13.33
**Valsartan**	6.67
**Clopidogrel**	3.32
**Metformin**	7.05
**Albendazole**	10.11
**Rupatadine**	5.42
**Miconazole**	11.00
**Gabapentin**	8.54
**Ezetimibe**	5.98
**Ascorbic acid**	16.67
**Uric acid**	18.57
**Magnesium sulfate**	80
**Copper sulfate**	53.33

^*^The tolerance limit is the maximum concentration in µg/mL of the interfering substance that caused a relative error (RE) less than 5% for the determination of the studied drugs

If the signal variation was less than 5%, we assumed that no interference had occurred. In general, the tolerance to the presence of foreign species is much higher than the concentration at which these compounds are often present with the analytes in pharmaceuticals (Table 4). Satisfactory tolerances were obtained due to the selectivity of the CQDs system towards the studied drugs, observing no interaction with other antihypertensive drugs and interfering agents.

### Mechanism of Quenching

**Fig. 4:**
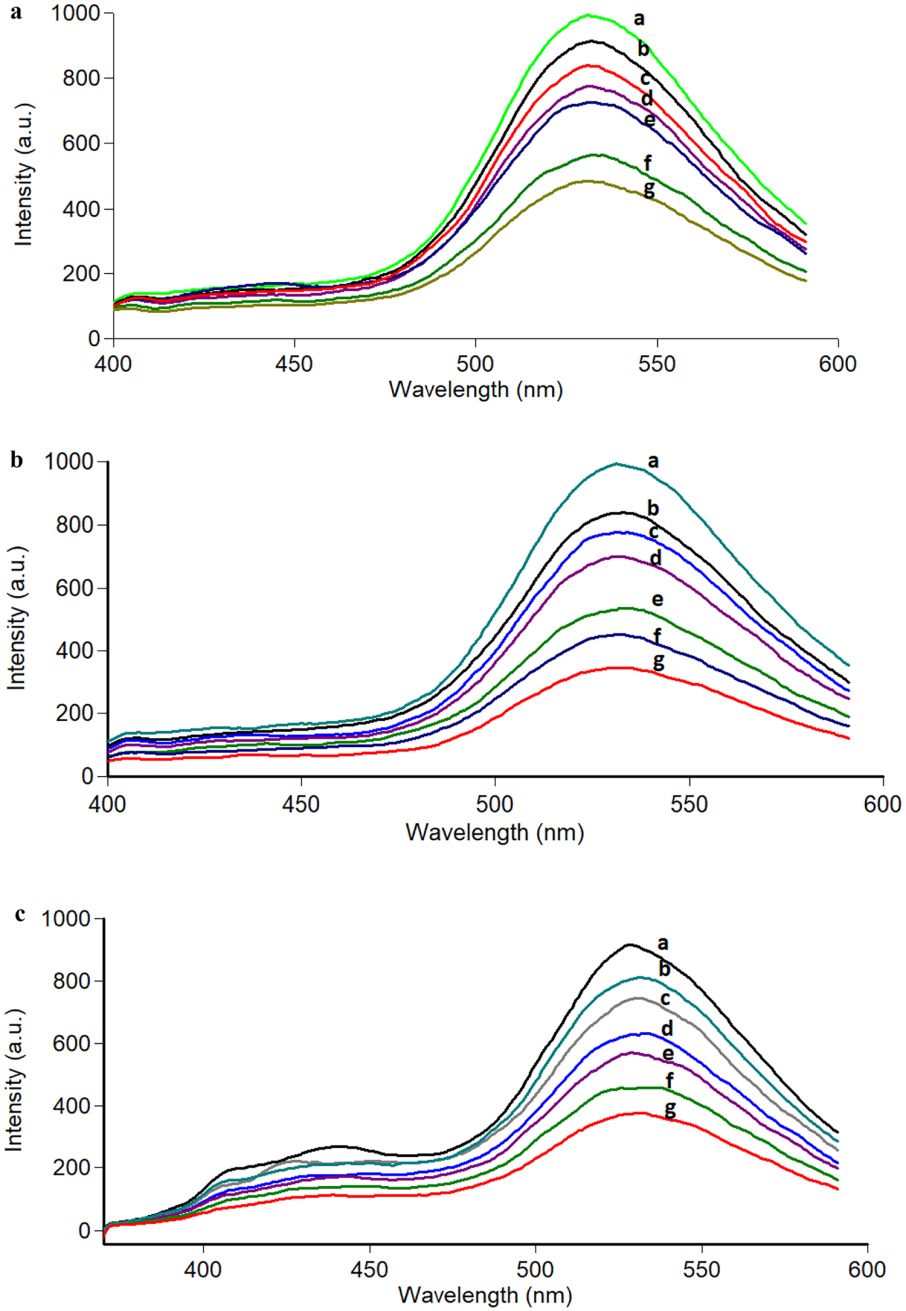
**(c):** Determination of the three drugs by the proposed method where a is the blank of CQDTs and b to g is the reaction product with different concentrations (0.5–20.0 µg/mL). a- Lercanidipine b- Nimodipine c- Nifedipine

The studied drugs were added to the synthesized CQDts, which exhibited fluorescence that was significantly quenched. The excitation and emission fluorescence spectra of CQDts at 325 and 528 nm, which were noticeably sensitive to each drug and gradually declined with increasing drug concentration, are shown in (Fig. 4). Fluorescence is known to be quenched through a variety of mechanisms, including fluorescence resonance energy transfer (FRET), inner filter effect (IFE), dynamic quenching, and static quenching [43].

**Fig. 5:**
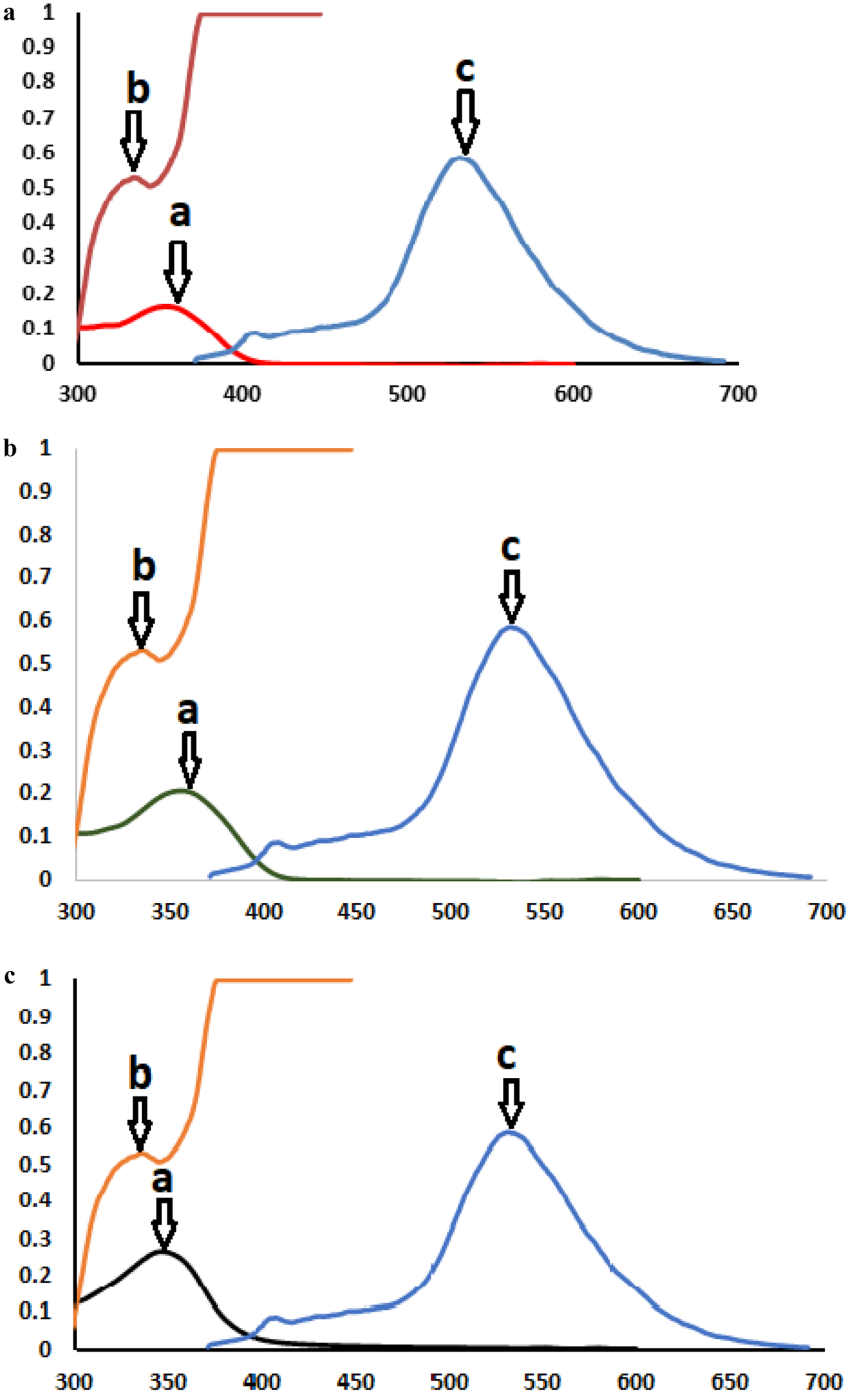
A co-plot showing the great overlap between the UV–Vis absorption spectrum of the three drugs and the fluorescence spectrum of the CQDs. **a** Lercanidipine **b** Nimodipine **c** Nifedipine

For each of the three drugs, the CQDts excitation spectra and UV absorbance spectra overlapped (Fig. 5), suggesting the possibility of IFE. To fully investigate the quenching mechanism, Eq. (1) was used in conjunction with the correction of the fluorescence intensity of the CQDts upon the addition of increasing concentrations of the quencher (lercanidipine, nimodipine, nifedipine).

1 $${\mathrm{F}}_{\mathrm{corr}}= {\mathrm{F}}_{\mathrm{obs}}\times {10}^{(\mathrm{Aex}+\mathrm{Aem})/2}$$

F_corr_ is the corrected fluorescence intensity after eliminating IFE from F_obs_, where F_obs_ is the observed fluorescence intensity. The terms A_ex_ and A_em_ relate to the quencher's absorbance at the fluorophore's (CQDts) excitation and emission wavelengths, respectively. The suppressed efficiency (% E) for the measured and corrected fluorescence intensity was then calculated using Eq. (2):

2 $$\mathrm{E }= \left[1-\frac{F}{F^\circ }\right]\times 100$$

**Fig. 6:**
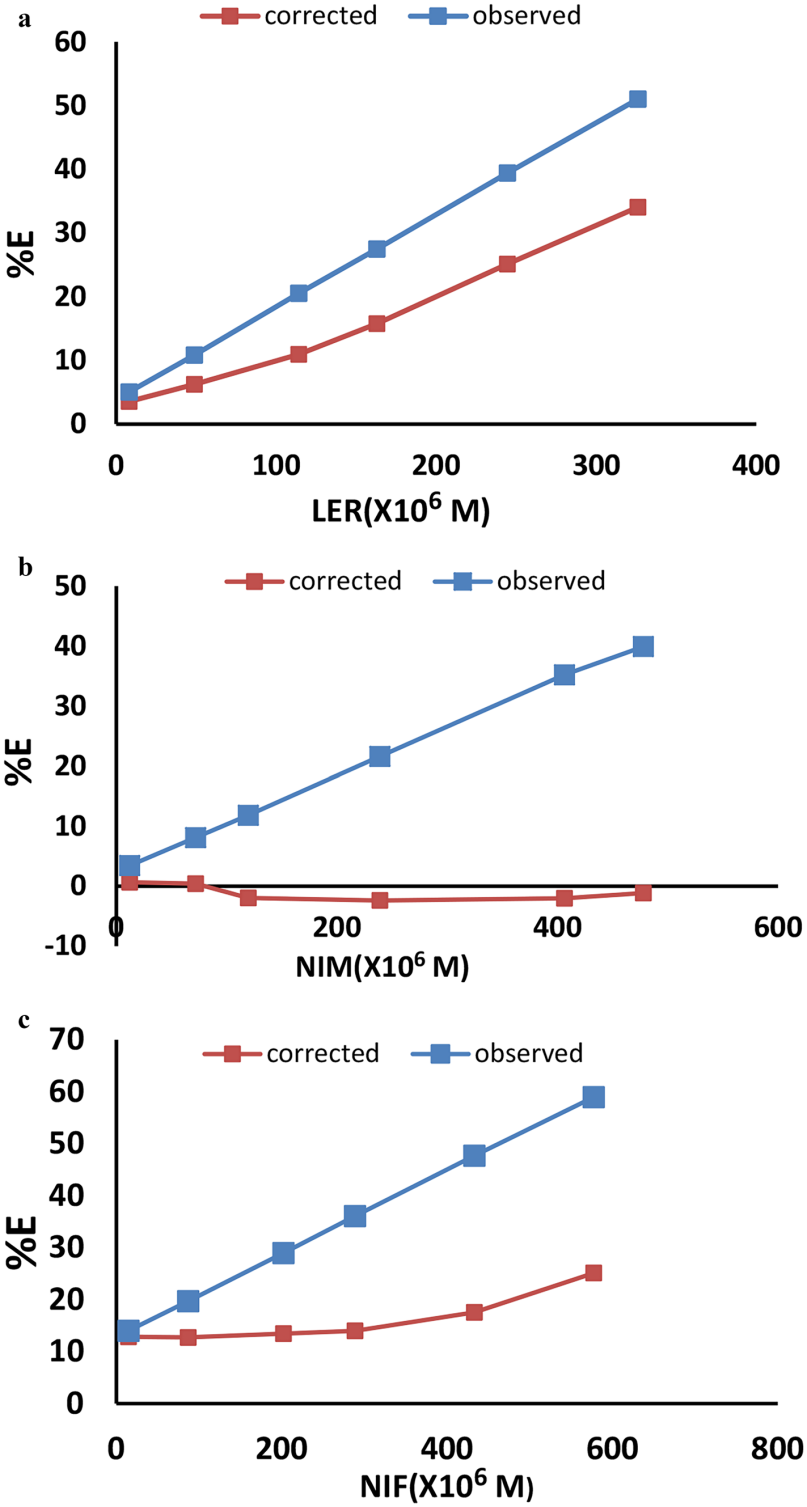
Suppressed efficiency of observed and corrected fluorescence of CQDs after addition of different concentrations of **a** Lercanidipine **b** Nimodipine **c** Nifedipine

Plotting % E of the observed and corrected fluorescence intensities of CQDts against the molar concentration of each of the three drugs indicated that IFE was the mechanism of quenching for NIM in this study (Fig. 6), while LER and NIF were found to have different mechanisms, requiring the use of Stern Volmer Eq. (3).

the Stern–Volmer plots were constructed by plotting F^o^/F versus [M] according to the Stern–Volmer equation [43]:

3 $${\mathrm{F}}^{\mathrm{o}}/\mathrm{F }=1+\mathrm{ Ksv }[\mathrm{M}]$$

{K_SV_ is the Stern–Volmer quenching constant and [M] is the molar concentration of each of LER and NIF}.

The formation of ground-state complexes provides static quenching, whereas the interaction of quencher molecules with the excited fluorophore results in dynamic quenching. These mechanisms can be found by examining the relationship between temperature and the quenching rate constant.

When the temperature rises during dynamic quenching, the quencher and fluorescent molecules are tempted to disperse and collide, increasing the quenching rate constant. The quencher/fluorophore ground-state complex becomes less stable at higher temperatures during static quenching, which results in a decrease in the quenching rate constant.

In this research, the effects of fluorescence quenching at 298, 308, and 318 K were examined. The experimental data were analyzed using the Stern–Volmer equation, and Ksv values were determined from the slope of the plots of F0/F versus [Q] (Fig. S5).

The results revealed a direct correlation between Ksv values and temperature (Table 5), demonstrating that reaction of lercanidipine and nifedipine with CQDts was IFE and dynamic interactions dominated the control of the fluorescence quenching process. additionally, KQ values were calculated and added to (Table 5) and were found to be lower than the maximum diffusion rate constant (2 × 10^10^) also for lercanidipine and nifedipine there was no difference between the UV vis spectra for the drugs with CQDts and the spectra for CQDts only (Fig. S6) which confirms that the mechanism of quenching was dynamic quenching.

**Table 5:** A summary of the Stern–Volmer parameters for the reaction of lercanidipine and nifedipine with CQDts

**Drug**	**Temperature (°k)**	**Stern–Volmer quenching constant (Ksv)**	**KQ**	**Correlation coefficient (r)**
**Lercanidipine**	298	0.018	180,000	0.9914
	308	0.0364	364,000	0.9984
	318	0.0478	478,000	0.9992
**Nifedipine**	298	0.0414	414,000	0.987
	308	0.116	1,160,000	0.9981
	318	0.1796	1,796,000	0.9989

## Conclusion

A new facile method was investigated for the determination of three pharmaceutically important CCBs, namely, lercanidipine, nimodipine, and nifedipine. This method is based on the quenching of QDs synthesized using ascorbic acid as a carbon source to generate high fluorescent carbon QDs and was optimized using a full factorial design.

The inner filter effect was recognized as a possible quenching mechanism between QDs and nimodipine, whereas the Inner filter effect and dynamic quenching were shown to be present with both lercanidipine and nifedipine. This approach enables the determination of the examined drugs in their pharmaceutical products and has been confirmed to have good linearity (0.5–20.0 µg/mL), low detection limits (0.11 ± 1.09 for lercanidipine, 0.10 ± 0.25 for nimodipine and 0.12 ± 0.71) and satisfactory recovery.

## Supplementary Information

Below is the link to the electronic supplementary material.

Supplementary file1 (DOCX 424 KB)

## Data Availability

All data analyzed during this study are included in this published article and raw data are available from the corresponding author on reasonable request.
