# Supplementary material for: A Design-assisted Spectrofluorometric Method Utilizing a One-pot Fluorescent Probe for the Quantitation of some Calcium Channel Blockers
Source: J Fluoresc. 2022 Dec 8;33(2):671–83. doi: 10.1007/s10895-022-03089-9 (PMC9957840; doi:10.1007/s10895-022-03089-9)
Supplement: Supplementary file 1 — Supplementary file1 (DOCX 424 KB) [file 10895_2022_3089_MOESM1_ESM.docx]

**SUPPLEMENTARY MATERIAL**

**Captions:**

**Table S1:** Assay results for the determination of the studied drugs in pure form by the proposed and comparison methods

**Figure S1:** FT-IR spectra of CQDts.

**Figure S2:** Typical TEM image of CQDts.

**Figure. S3:** 2^3^ FFD half normal plots and Pareto charts plots of the effects on the chromatographic responses at alpha=0.05 for a- Lercanidipine b- Nimodipine c- Nifedipine.

**Figure. S4:** 2^3^ FFD interaction plots and main effect plots for chromatographic responses by data means type a- Lercanidipine b- Nimodipine c- Nifedipine.

**Figure. S5:** Stern-Volmer plots (at different temperature settings) for a- Lercanidipine

b- Nifedipine

**Figure. S6:** UV-vis spectra for CQDts only and after the addition of 10 µg/mL of a-lercanidipine b-nifedipine

**Table S1:** Assay results for the determination of the studied drugs in pure form by the proposed and comparison methods

| **Compound** | **Proposed Method** | | | **Comparison methods** [5,11,25] |
| --- | --- | --- | --- | --- |
|  | Amount taken  (µg/mL) | Amount found  (µg/mL) | % Found | % Found |
| Lercanidipine | 0.5 | 0.507 | 101.72 | 99.20 |
|  | 3 | 2.944 | 98.05 | 100.82 |
|  | 7 | 7.048 | 99.61 | 99.73 |
|  | 10 | 9.998 | 101.69 |  |
|  | 15 | 15.043 | 99.74 |  |
|  | 20 | 19.959 | 99.90 |  |
| Mean | 100.12 | | | 99.92 |
| ± S.D. | 1.40 | | | 0.83 |
| t-test | 0.16 (2.37) | | | |
| F-test | 1.72 (19.3) | | | |
| Nimodipine | 0.5 | 0.499 | 99.78 | 100.54 |
|  | 3 | 3.000 | 100.02 | 99.47 |
|  | 5 | 4.991 | 99.82 | 100.18 |
|  | 10 | 9.994 | 99.94 |  |
|  | 17 | 17.069 | 100.40 |  |
|  | 20 | 19.947 | 99.73 |  |
| Mean | 99.95 | | | 100.06 |
| ± S.D. | 0.25 | | | 0.54 |
| t-test | 0.29 (2.37) | | | |
| F-test | 4.67 (5.79) | | | |
| Nifedipine | 0.5 | 0.5024 | 100.48 | 100.79 |
|  | 3 | 2.9612 | 98.71 | 99.22 |
|  | 7 | 6.9803 | 99.72 | 100.26 |
|  | 10 | 10.0538 | 100.54 |  |
|  | 15 | 15.0660 | 100.44 |  |
|  | 20 | 19.9362 | 99.68 |  |
| Mean | 99.93 | | | 100.09 |
| ± S.D. | 0.71 | | | 0.80 |
| t-test | 0.28 (2.37) | | | |
| F-test | 1.27 (5.79) | | | |

***N.B.*** ^*^Mean of three determinations.

^a^ The values between parentheses are the tabulated t and F values at *P* = 0.05[43].

**
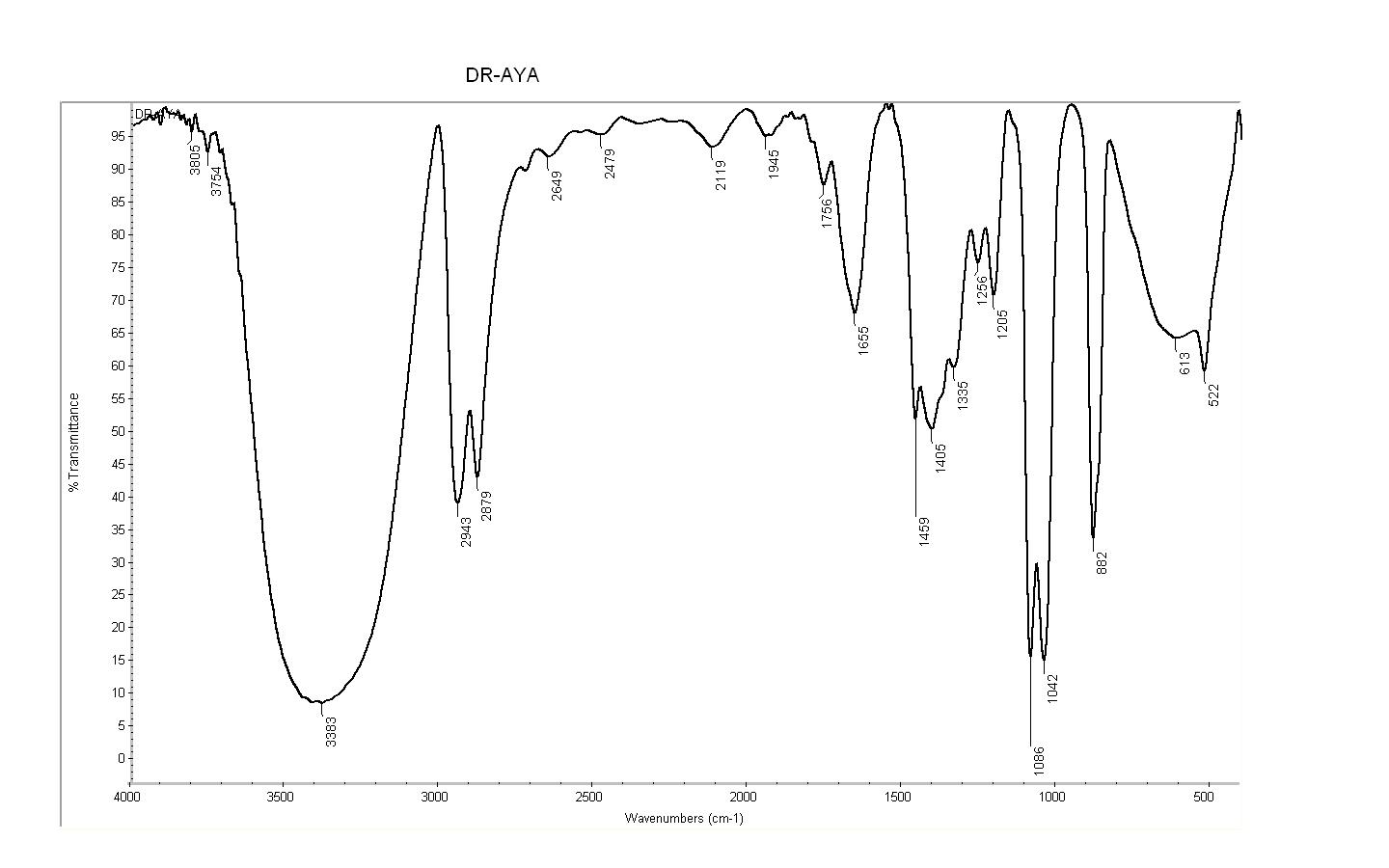
**

**Figure S1**: FT-IR spectra of CQDts.


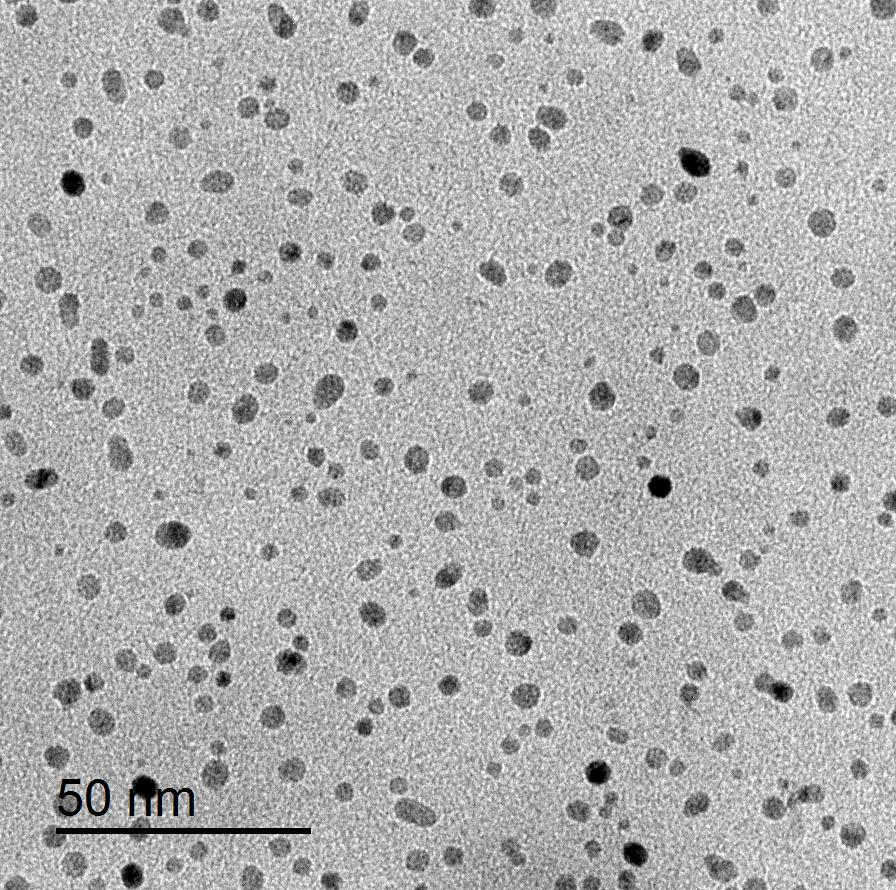


**Figure S2:** Typical TEM image of C CQDts.

**Figure. (S3-a):** 2^3^ FFD half normal plots and Pareto charts plots of the effects on the chromatographic responses at alpha=0.05 for Lercanidipine

**Figure. (3S-b):** 2^3^ FFD half normal plots and Pareto charts plots of the effects on the chromatographic responses at alpha=0.05 for Nimodipine

**Figure. (S3-c):** 2^3^ FFD half normal plots and Pareto charts plots of the effects on the chromatographic responses at alpha=0.05 for Nifedipine

**Figure. (S4-a):** 2^3^ FFD interaction plots and main effect plots for chromatographic responses by data means type for Lercanidipine.

**Figure. (S4-b):** 2^3^ FFD interaction plots and main effect plots for chromatographic responses by data means type for Nimodipine.

**Figure. (S4-c):** 2^3^ FFD interaction plots and main effect plots for chromatographic responses by data means type for Nifedipine.

**Figure. (S5-a):** Stern-Volmer plots (at different temperature settings) for Lercanidipine

**Figure. (S5-b):** Stern-Volmer plots (at different temperature settings) for Nifedipine

**Figure. S6-a:** UV-vis spectra for CQDts only and after the addition of 10 µg/mL of lercanidipine

b

**Figure. S6-b:** UV-vis spectra for CQDts only and after the addition of 10 µg/mL of nifedipine
